# The Transcription of *ZIP9* Is Associated With the Macrophage Polarization and the Pathogenesis of Hepatocellular Carcinoma

**DOI:** 10.3389/fimmu.2022.725595

**Published:** 2022-03-15

**Authors:** Yingying Gou, Dan Yang, Taikun Tian, Xingguo Zhu, Raorao Zhang, Jiaqi Ren, Dezhen Tu, Yi Luo, Yuqing Miao, Huan Zhao, Yu Wang, Bin Wei

**Affiliations:** ^1^ School of Life Sciences, Shanghai University, Shanghai, China; ^2^ Shanghai Engineering Research Center of Organ Repair, Shanghai University, Shanghai, China; ^3^ State Key Laboratory of Virology, Wuhan Institute of Virology, Chinese Academy of Sciences, Wuhan, China; ^4^ University of Chinese Academy of Science, Beijing, China; ^5^ State Key Laboratory of Cell Biology, Shanghai Institute of Biochemistry and Cell Biology, Center for Excellence in Molecular Cell Science, Chinese Academy of Sciences, Shanghai, China; ^6^ Department of Head & Neck Surgery, Fudan University Shanghai Cancer Center/Cancer Institute, Shanghai, China; ^7^ Department of Oncology, Shanghai Medical College, Fudan University, Shanghai, China; ^8^ Department of Respiration Medicine, Affiliated Nantong Hospital of Shanghai University, Nantong, China; ^9^ Cancer Center, Shanghai Tenth People’s Hospital, School of Medicine, Tongji University, Shanghai, China

**Keywords:** *Zip9_1_*, hepatocellular carcinoma, macrophages polarity, *Zip2*, ZIPs

## Abstract

Hepatocellular carcinoma (HCC) is one of the most common digestive system cancers (DSCs) with a poor prognosis. Zinc‐regulated transporter (ZRT)/iron‐regulated transporter (IRT) like protein transporters (*ZIPs*) encode membrane transport proteins, which are responsible for the absorption of zinc and play important roles in the pathogenesis of various human cancers. Tumor-associated macrophages (TAMs) are important participants in the regulation of tumor microenvironment and the development of HCC. Individual role of each *ZIP* involved in hepatocarcinogenesis remains elusive. In this study, the transcription patterns of *ZIPs* in the DSCs were screened firstly through GEPIA2 database. Interestingly, the analysis of the DSCs data showed the distinct mRNA levels of *ZIPs* between DSCs tissues and healthy controls. Notably, the transcription levels of *ZIP2*, *ZIP5*, *ZIP8*, *ZIP9* and *ZIP14* were decreased significantly in the tissues of human liver cancer compared to paracarcinoma liver tissues. To further confirm the mRNA transcriptional changes of *Zips* in HCC, N-Nitrosodiethylamine (DEN) combined with carbon tetrachloride (CCl_4_) inducing mouse model of HCC were established. Consistently, the mRNA levels of *Zip2*, *Zip9*, and *Zip14* in liver tissues of HCC induced mice were also decreased compared with the healthy controls. In addition, mouse peritoneal elucidated macrophages (PEMs)-derived M1/M2 macrophages *in vitro*, as well as human patients of HCC-derived TAMs, were used to examine the transcription levels of *ZIPs*. Our results showed that both *Zip2* and *Zip9* were up-regulated in M2-polarized macrophages. *Zip2* transcript was also up-regulated M1-polarized macrophages, but *Zip9* was slightly down-regulated. TAMs generated from human liver cancer tissues also displayed a decrease in *ZIP9* transcription compared to paracarcinoma tissues. To further explore the role of Zip9 in M1/M2 polarization, the siRNA knockdown results revealed that *Zip9*, but not *Zip2*, could promote M2 macrophage polarization and impair M1 macrophage polarization. Mechanistically, *Zip9* enhances phosphorylated STAT6 to promote M2 macrophage polarization but suppresses the phosphorylation of IκBα/β to inhibit M1 macrophage polarization. Together, our results indicate that *ZIP9* may involve in macrophages polarity in HCC development and may be a potent new biomarker for the diagnosis of HCC.

## Introduction

Digestive system cancers (DSCs, including esophageal cancer, gastric cancer, colorectal cancer, cholangiocarcinoma, hepatocellular carcinoma, and pancreatic cancer) are common malignant cancers and the cause of death worldwide (1). Although the survival rates of these cancers have been greatly improved in recent years due to the advancement of early diagnosis and treatment strategies, the incidence and mortality of these cancers have increased (2, 3). Among these DSCs, hepatocellular carcinoma (HCC) is the sixth most common primary malignant tumor in the world, and it is the fourth leading cause of human death (4). It is well documented that the level of zinc was significantly lower in the human (hepatoma tissue) compared with surrounding “normal” or cirrhotic tissue (5). Zinc is the second essential trace element and plays an important role in all organisms, such as maintaining the stability and function of many metalloenzymes involved in protein synthesis, protein catabolism, energy metabolism, and RNA and DNA synthesis (6). An association between lower systemic zinc levels and DSCs has been demonstrated in various human disorders, such as hepatitis, colorectal cancer and gastric cancer (7–9).

The homeostasis of zinc in the body is mainly regulated by zinc transporters (10). These zinc transporters can be divided into two families, zinc transporters (CDF/ZnTs) and zinc‐regulated transporter (ZRT)/iron‐regulated transporter (IRT)‐like protein transporters (ZIPs). ZnTs are encoded by 10 genes and are responsible for zinc efflux, while ZIPs are encoded by 14 genes and are responsible for the influx of zinc into the cytoplasm (10). Multiple studies had highlighted the key functions of *ZIP* genes in DSCs, like colorectal cancer, esophageal carcinoma, pancreatic cancer, and hepatocellular carcinoma (11–15). Besides, emerging evidence indicated that *ZIP* genes were closely correlated with the prognosis of patients of DSCs. Some specific *ZIP* genes had been suggested to serve as cancer diagnostic or prognostic biomarkers. Lou et al. evaluated the prognostic values of *ZIP* family genes in gastric cancer (GC) patients by using a variety of online databases. Their results indicated that the high mRNA levels of *ZIP7*, *ZIP11*, *ZIP14* were associated with better overall survival (OS), while *ZIP1*, *ZIP2*, *ZIP3*, *ZIP4*, *ZIP5*, *ZIP6*, *ZIP8*, *ZIP9*, *ZIP12*, *ZIP13* were significantly related with worse OS in GC patients (16). In addition, ZIP3 has been reported to be downregulated in the early stage of pancreatic cancer (17). Xiao et al. reported that overexpression of ZIP4 increased cell migration and invasion in HepG2 cells (18). Zhu et al. found that ZIP4 was overexpressed in human pancreatic cancer tissues and cell lines (19). ZIP4 was also significantly enhanced in esophageal cancer (ESCC) specimens, which could serve as a novel prognostic to promote ESCC progression (20). Furthermore, ZIP5 was overexpressed in human esophageal cancer tissue in regions of high incidence, and downregulation of ZIP5 inhibited the proliferation, migration, and invasion of ESCC *in vivo* (21). Also, *ZIP6* gene was up-regulated in ESCC tissue, high tumorous ZIP6 expression was significantly correlated with shorter OS (14). All these researches indicate that *ZIPs* function in the development of DSCs. However, to our knowledge, the relevant studies regarding the total transcription patterns of *ZIP* genes in the development of HCC are still limited.

Macrophages can be characterized by a high diversity of cell surface agents, transcriptional profiles, and different self-local environment-derived stimuli can induce the polarization of macrophage phenotypes (22). M1 macrophages are activated by interferon-γ (IFNγ) produced by CD8^+^ T cells and natural killer (NK) cells (23, 24). They are also activated by PAMPs, such as lipopolysaccharide (LPS), cytoplasmic receptors (25), the pro-inflammatory cytokines and chemokines (26). Those activated M1 macrophages can induce the synthesis of reactive oxygen species (ROS) and the release of nitric oxide (NO) (27). Macrophages polarized to M2 phenotype (induced by IL-4 and IL-13) can promote HCC progress (28). It has been shown that M2-polarized tumor associated macrophages (TAMs) influence HCC cells *via* the IL-6/STAT3 signaling pathway (29, 30). It also has been reported that HCC deteriorates on account of the upregulation of protein in M2 TAMs (31). More importantly, it has been elucidated that many transcription factors are involved in regulating M1/M2 polarization of macrophages, NF-κB promotes M1 polarization and STAT6 is essential to enhance the expression of M2 related genes (32, 33) Although some genes in *Zip* family are illustrated to be associated with the function of macrophages (34), the effect of *Zips* on the polarization of macrophages is poorly understood.

In this article, we focused on the transcriptome analysis of total *ZIPs* in human HCC patients and total *Zips* in DEN-induced HCC mice and their roles in polarized-macrophages *in vitro* and TAMs of HCC patients *in vivo*. Interestingly, our results showed that the mRNA level of *ZIP9* (*Zip9*) was significantly down-regulated in tumors of both human and mouse HCC. Moreover, *Zip9* knockdown specifically reduced M2 *via* the IL-4/STAT6 signaling pathways but enhanced M1 macrophage polarization *via* the NF-κB signaling pathways in response to inflammatory stimuli. Importantly, mRNA expression of *ZIP9* was also down-regulated in human HCC-derived TAMs. This study may provide new insight into ZIP9 in HCC development and help to develop potent strategies for the diagnosis of patients with HCC.

## Materials and Methods

### Mice

C57BL6 background mice were purchased from the Model Animal Research Center (Nanjing University, Nanjing, China). All animals were housed in animal facilities at the Shanghai University under a standard 12‐h light/dark cycle with access to chow and water ad libitum. Experiments were conducted in accordance with the ethical statement.

### DEN and CCl_4_- Induced Mouse Model of HCC

6-8 weeks of age male C57BL/6J mice were treated with DEN intraperitoneally (i.p., N0258‐1G; Sigma, St. Louis, MO, USA) at a dose of 100 mg·kg^-1^ or with vehicle alone (normal saline, i.p., referred as control). Four weeks later, mice were weekly i.p. injected with CCl_4_ (0.5 ml/kg, dissolved in olive oil) for 12 weeks. CCl_4_ was diluted 1: 9 in olive oil (A502795-0100, Sangon Biotech). The mice of the control group were treated with olive oil alone. Finally, mice were sacrificed for collection of liver tissues.

### Collecting PEMs and Inducing M1/M2 Macrophages

To obtain peritoneal elucidated macrophages (PEMs), mice were injected i.p. with 3 ml sterile 3% Brewer thioglycollate medium; 3 days later, PEMs were harvested. PEMs were maintained in complete DMEM supplemented with 10% (vol/vol) FBS and penicillin/streptomycin (100 U/ml) (Sigma-Aldrich, Taufkirchen, Germany) at 37°C and 5% CO_2_. Post 24hrs of cell culture, media was replaced with fresh media containing either lipopolysaccharide (LPS; 1μg/ml; Sigma-Aldrich, L6529) and interferon γ (IFNγ; 100ng/ml; Peprotech, 315-05) to induce M1 macrophages or a combination of IL-4 (20ng/ml; Peprotech, 214-14) and IL-13 (20ng/ml; Peprotech, 210-13) to induce M2 macrophages.

### RNA Extraction and Quantitative Real-Time Polymerase Chain Reaction (RT-PCR)

Total RNA was isolated from cells or tissues using Trizol (Takara). The RNA concentration and purity were determined spectrophotometrically. And cDNA was synthesized using approximately 1μg of total RNA and the reverse transcriptase M-MLV (Takara Bio, Shiga, Japan). Quantitative real-time PCR (RT-PCR) was carried out using 2× SYBR green (Takara Bio, Shiga, Japan) with specific primers for ZIPs (1–14), YM1, ARG1, IL6 and β-Actin (**Table 1**) in a C1000 thermal cycler (BIO-RAD CFX96, Hercules, CA, USA). β-Actin was used for normalization. The relative mRNA expression was calculated using the 2^−ΔCT^ method or 2^-ΔΔCT^ method.

**Table 1:** Primer sequences used for real time RT-PCR.

Gene name	Forward primer	Reverse primer
mouse		
*ß-Actin*	CCTGGCACCCAGCACAAT	GGGCCGGACTCGTCATACT
*Il-6*	TGTATGAACAACGATGATGCACTT	ACTCTGGCTTTGTCTTTCTTGTTATCT
*Arg1*	CTCCAAGCCAAAGTCCTTAGAG	AGGAGCTGTCATTAGGGACATC
*Ym1*	CAGGTCTGGCAATTCTTCTGAA	GTCTTGCTCATGTGTGTAAGTGA
*Fizz1*	CCAATCCAGCTAACTATCCCTCC	ACCCAGTAGCAGTCATCCCA
*Inos*	GGAGTGACGGCAAACATGACT	TCGATGCACAACTGGGTGAAC
*Il4*	GGTCTCAACCCCCAGCTAGT	GCCGATGATCTCTCTCAAGTGAT
*Il10*	CAGTACAGCCGGGAAGACAA	AGGCTTGGCAACCCAAGTAA
*Tgfb*	ACCATGCCAACTTCTGTCTG	CGGGTTGTGTTGGTTGTAGA
*Tnfa*	AGTGACAAGCCTGTAGCCC	GAGGTTGACTTTCTCCTGGTAT
*Zip1*	ACTACCTGGCTGCCATAGA	TGAACTCTTGCAGTGGGAAC
*Zip2*	CTGGAGGGAATTGAGTCAGAAA	AAGCAGCATCACGAGAAGAA
*Zip3*	CCTGCAGTGAGGGACAAG	GGTAGTCGGTGCTGATGTG
*Zip4*	GGACCAGCTCAGTCAAACA	GACCGAACACAGCACAGA
*Zip5*	TGCTAGAGAACACACTAGGACT	CAGGGTTTGGTTCTCCAAGAT
*Zip6*	CGTACTCACACTGATCAAGCA	TGCTTCTTGCTCTCCACATC
*Zip7*	GACATGGACACTCCCACAG	GCGACAATCCCACTGAGAA
*Zip8*	TCTAAGAAAGCACAACGCAAAG	AGGAGAGAGGCCAGATTGATA
*Zip9*	TCATTCCTTTGGCTGTTAATTTCTC	GCAGTTCCACAGAGAAGACC
*Zip10*	ACTCTGGTTCCTGAAGATAAGAC	GCAGACTAATGACGGTGATAGA
*Zip11*	CGGAGAGTGAACTTTCCATCC	CAGTAGCTGCCACCTTCTTC
*Zip12*	AGTACTTTGGCACTTCCAGTAG	CAGATTCCCTCTGCAGAATCTTA
*Zip13*	GAAGATGTTCCTCAACAGCAAG	CAGACAGTGGCCTCCATT
*Zip14*	TTTCCCAGCCCAAGGAAG	CAAAGAGGTCTCCAGAGCTAAA
human		
*ACTIN*	CATGTACGTTGCTATCCAGGC	CTCCTTAATGTCACGCACGAT
*AFP*	TGCAGCCAAAGTGAAGAGGGAAGA	CATAGCGAGCAGCCCAAAGAAGAA
*GPC3*	AGAGGCCTTTGAAATTGT	AAATACTTTCAGGTCACGTC
*MST1*	CAAGCGGAATACAGTGATAGG	GTGGGAGGAGGATTTGTAGG
*TGFB*	CCCAGCATCTGCAAAGCTC	GTCAATGTACAGCTGCCGCA
*IL6*	GGATTCAATGAGGAGACTTGC	GTTGGGTCAGGGGTGGTTAT
*IL1B*	TGGTGGTGAAGACACCAGTAG	TGGAGAACACCACTTGTTGCT
*TNFA*	AGGCGCTCCCCAAGAAGACA	AAGTGCAGCAGGCAGAAGAG
*ZIP1*	CGGCCAGGAGCTAACCATGAAG	GCCCACCATTCACTGTTCCCAG
*ZIP2*	ACTCTGGGCTGTGGCCTTACTC	AAGCCCAGGGAGATGATGAGC
*ZIP3*	TGGTTGGGCTCGGTAGCACATC	GTCACTGCAGGGCCAAACCATC
*ZIP4*	CAACAGCTCCAGTGTGTGGGAC	GCTCAGGAAGGTCTGCAGGATG
*ZIP5*	TCATTGGCTGACCACCTGAATG	AGCAAGGGCCGTAGTAGACGAG
*ZIP6*	CATGGCATGGGCATCCAGGTTC	TCAAAGTCCCAACGGCCAGTGC
*ZIP7*	CAGGACCTGGATGCTGTCACTC	CGACAAGAAAGGCAACAATTCC
*ZIP8*	CTGCCATCAATGGTGTGACATG	CCCTGAAGGAGAGACAAGGTGC
*ZIP9*	TGCTGGCCTTCTCTGTGGAAC	TGCTAGACCTTGCTGCTTCTGG
*ZIP10*	GCATAATCGGGTCCACAAACC	AACAATGCAGGGCAAAGGTATG
*ZIP11*	CGGCATCTGCTACCTTTGAGAG	ATGATGTCGTCCATGACCACG
*ZIP12*	CGAATACCCTCCGCCTATCAG	TGCTGGATGATCCCTGGACTC
*ZIP13*	CTCTTGGGCAATGTGTTTCTGC	GACTTTGATGCTCCGGACCAC
*ZIP14*	CTCTGTGTGACCGTCATCTCCC	AAGCGACTCAGAGGCATAATGG

### Patients and Tissue Samples

A total of 9 patients with histopathologically confirmed liver cancer were collected from Shanghai Zhongshan Hospital (Shanghai, China). All the samples were obtained *via* surgical resection or liver transplantation procedures. The diagnosis of liver cancer was performed following the WHO Classification of Cancers of the Digestive System (1). All the tissue samples were reviewed by 2 independent experienced pathologists. The study protocol was approved by the Ethical Committee of Shanghai Zhongshan Hospital. Written informed consent was obtained from all patients.

### SiRNA Transfection

The synthesized siRNA was transfected into murine macrophages using Lipofectamine RNAiMAX (Invitrogen). siRNA sequences used for transfection was shown in **Table 2**.

**Table 2:** siRNA sequence used for transfection.

Gene	Sense	Anti-sense
Zip2#1	GGAAAUUCUUCUCGUGAUG UU	CAUCACGAGAAGAAUUUCC UU
Zip2#2	CAUAGCCGCUGGCACGUUUUU	AAACGUGCCAGCGGCUAUG UU
Zip9#1	GCAGCAGAAAUAUCAUCUG U	CAGAUGAUAUUUCUGCUGC UU
Zip9#2	UCAUUCCUUUGGCUGUUAA UU	UUAACAGCCAAAGGAAUGA UU

### Tissue Paraffin Section Preparation and HE Staining

The liver tissues were fixed in 4% paraformaldehyde or 10% neutral formaldehyde overnight then treated with different alcohol for dehydration, xylene solution for transparency, and finally embed the tissue in paraffin, used a microtome to cut the tissues into 3.5μm slices for HE staining and IHC staining. For HE staining, briefly, after deparaffinization and rehydration, sections were stained with hematoxylin solution for 5 min followed by dipping in 1% acid ethanol (1% HCl in 70% ethanol) and then rinsed with distilled water. Then the sections were stained with eosin solution for 3 min and followed by dehydration with graded alcohol and clearing in xylene. The slides were then examined and photographed under Olympus BX53 fluorescence microscope (Tokyo, Japan).

### Immunohistochemistry

Paraffin-embedded liver tissue slides were kept at 60°C for 24 h, dewaxed with xylene and hydrated by gradient ethanol (100%-70%). The slides were incubated in a 1× antigen retrieval solution (YEASEN, 36310ES60) and an endogenous peroxidase blocker (ZSGB, SP-9000). Then the slices were incubated with goat serum working solution (ZSGB, SP-9000) for 10-15 min, and anti-CD68 primary antibody (CST, 76437T) at 4°C overnight, and finally washed with 1× PBS. The slides were rinsed with 1× PBS 3 times and incubated with the corresponding secondary antibody (ZSGB, SP-9000) for 30 min followed by 3, 3-diaminobenzidine (DAB) and hematoxylin staining, respectively. The slides were then examined and photographed using an Olympus BX53 fluorescence microscope (Tokyo, Japan).

### Western Blot

Cells were lysed in RIPA buffer (50 mM Tris-HCl at pH 7.4,150 mM NaCl, 1% Triton X-100, 1% sodium deoxycholate, 0.1% SDS, 1 mM EDTA) with protease and phosphatase inhibitors, followed by denaturation at 99 °C for 10 min. Then the protein samples were separated by SDS-PAGE and transferred onto polyvinylidene fluoride (PVDF) membranes (Millipore). Next, the PVDF membranes were blocked with 5% bovine serum albumin for 1 h and incubated with the primary antibodies overnight, including rabbit anti-STAT6 (1:1000, CST, 5397), rabbit anti-p-STAT6 (1:1000, CST, 9361), rabbit anti-p-p65 (1: 1000, CST, 3033), rabbit anti-IKKα/β (1:1000, CST, 2697) and rabbit anti-tubulin (1:5000, ProteinTech, 11224-1-AP). After that, the PVDF membranes were disposed of with the relevant secondary antibodies (1:5000) for 1 h at room temperature and observed using the ECL kit chemiluminescence reagents (Millipore, Billerica, MA, USA). The signals of protein bands were detected with the Chemidoc detection system and quantified using Quantity One software (Bio-Rad).

### Flow Cytometry and FACS

Livers of mice were harvested and pooled from 6-8 week C57BL/6J WT mice. Livers of human were collected from HCC patients in Shanghai Zhongshan Hospital (Shanghai, China). Red blood cells lysis was performed using ACK buffer. The panel of antibodies used in the experiments included APC-anti-F4/80 (Invitrogen, 17-4801-82), FITC-anti-CD11b (Invitrogen, 11-0112-82), APC-anti-CD11B (ThermoFisher, 11-0118-42), APC-anti-CD14 (ThermoFisher, 17-0149-42). Flow cytometry was performed using BD LSR II and BD FACS Aria III flow cytometers (BD Bioscience), and data were analyzed with FlowJo software (TreeStar, Ashland, OR).

### Statistics

Bioinformatics analysis of gene expression and Correlation analysis are available at the Gene Expression Profiling Interactive Analysis website (http://gepia2.cancer-pku.cn/#survival). Statistical data analysis was performed with Graph pad Prism 9.0 software. Data represent mean ± SD. Statistical significance was determined with 2-tailed unpaired Student’s t test between 2 groups. One-way or 2-way ANOVA with Holm-Sidak correction or with Newman-Keuls correction were used for multiple comparisons. Correlations were done using Pearson’s or Spearman’s tests. *P*< 0.05 was considered statistically significant (*P < 0.05, **P < 0.01, ***P < 0.001, and ****P < 0.0001).

## Results

### *ZIPs* in DSCs Show Distinct Transcription Patterns in Samples From GEPIA2 Database

**Figure 1:**
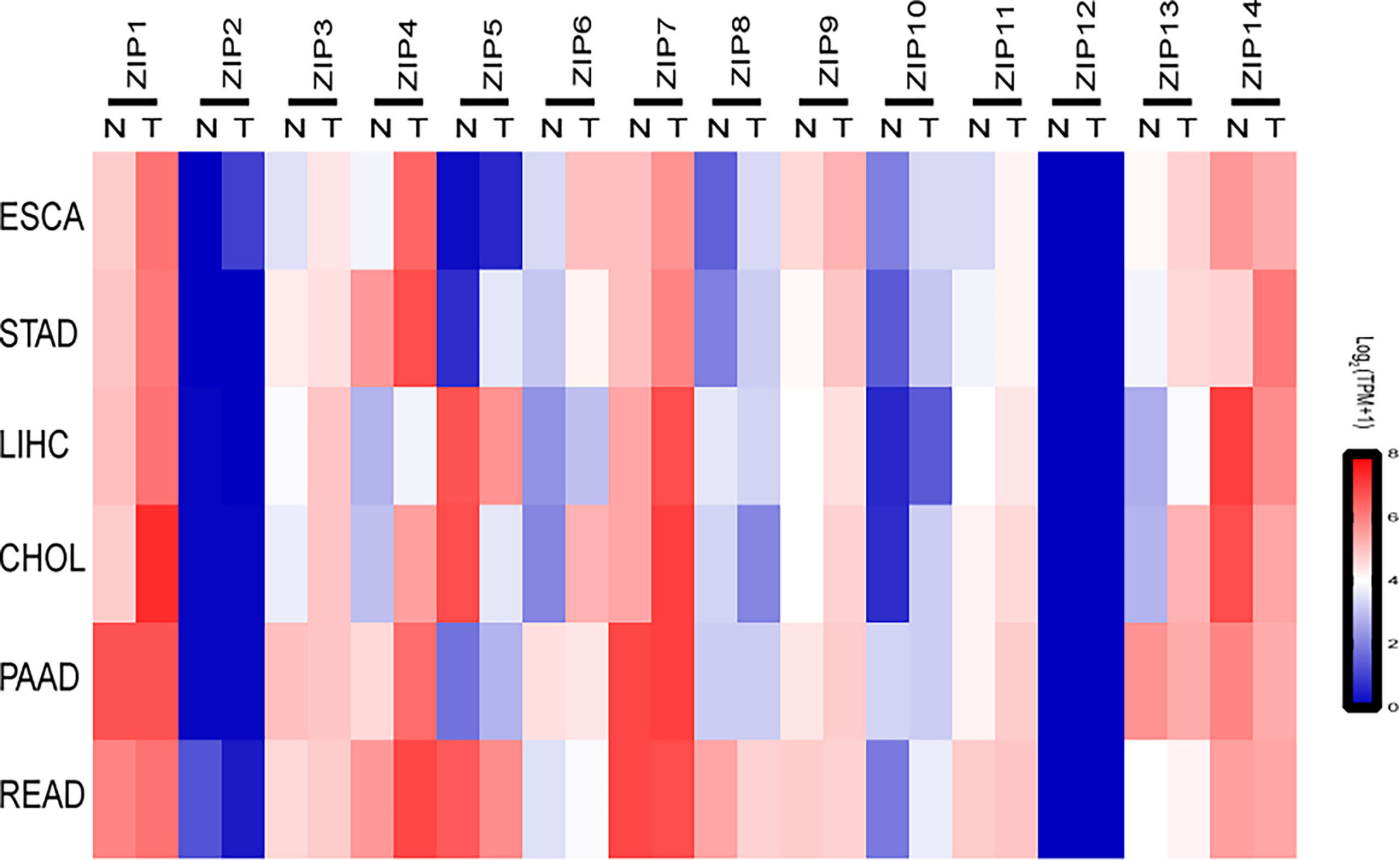
The transcription profiles of *ZIPs* in various cancers of the digestive system. Heat-map depicting the RNA expression profiles of *ZIPs* in a variety of human digestive system cancers based on RNA sequencing data from Gene Expression Profiling Interactive Analysis website (GEPIA2). Data were obtained from cancer patients (T) and healthy controls (N), and each sample was normalized by log_2_ (TPM+1). The red and blue regions represent the higher and the lower expression levels, respectively (TPM, transcripts per million; ESCA, Esophageal carcinoma; STAD, Stomach adenocarcinoma; LIHC, liver hepatocellular carcinoma; CHOL, Cholangiocarcinoma; PAAD, Pancreatic adenocarcinoma; READ, Rectum adenocarcinoma).

To explore the roles of *ZIPs* in DSCs, data were retrieved from patients with DSCs in the Gene Expression Profiling Interactive Analysis (GEPIA) and 72 normal samples from The Cancer Genome Atlas (TCGA) databases, we found except *ZIP12*, almost all *ZIP* genes had obviously different transcription patterns between tumor tissues and normal tissues (**Figure 1**). Our results showed that the transcription levels of five *ZIPs* were significantly elevated in cancer of esophageal carcinoma (ESCA). Seven *ZIPs* showed remarkable transcription increase in cancer of stomach adenocarcinoma (STAD). Seven *ZIPs* were significantly up-regulated in cancer tissues of cholangiocarcinoma (CHOL). Two *ZIPs* in rectum adenocarcinoma (READ) were notably down-regulated in cancers. Also, four *ZIPs* in liver hepatocellular carcinoma (LIHC) were substantially increased in cancers. Two *ZIPs* were significantly decreased in tumors of LIHC (**Table 3**). These data suggest that *ZIPs* have different transcription profiles in different DSCs, which implies their un-neglected physiological functions in the pathology of various DSCs.

**Table 3:** Summary of *ZIPs* transcription levels in human digestive system cancers (T) and healthy controls (N).

T vs N	ESCA	STAD	LIHC	CHOL	PAAD	READ
T>N	ZIP1*	ZIP1*	ZIP1*	ZIP1*	*ZIP4 ^ns^*	*ZIP1 ^ns^*
	*ZIP2 ^ns^*	*ZIP3 ^ns^*	ZIP3*	ZIP3*	*ZIP5 ^ns^*	*ZIP3 ^ns^*
	*ZIP3 ^ns^*	ZIP4*	*ZIP4 ^ns^*	ZIP4*	*ZIP7 ^ns^*	ZIP4*
	ZIP4*	ZIP5*	*ZIP6 ^ns^*	ZIP6*	*ZIP9 ^ns^*	*ZIP6 ^ns^*
	*ZIP5 ^ns^*	ZIP6*	ZIP7*	ZIP7*	*ZIP11 ^ns^*	ZIP10*
	ZIP6*	*ZIP7 ^ns^*	*ZIP9 ^ns^*	*ZIP9 ^ns^*		
	*ZIP7 ^ns^*	ZIP8*	*ZIP10 ^ns^*	ZIP10*		
	ZIP8*	*ZIP9 ^ns^*	*ZIP11 ^ns^*	*ZIP11 ^ns^*		
	*ZIP9 ^ns^*	ZIP10*	ZIP13*	ZIP13*		
	ZIP10*	*ZIP11 ^ns^*				
	*ZIP11 ^ns^*	*ZIP13 ^ns^*				
		ZIP14*				
T<N	*ZIP14 ^ns^*	*ZIP2 ^ns^*	*ZIP2 ^ns^*	ZIP5*	*ZIP3 ^ns^*	*ZIP2 ^ns^*
			ZIP5*	ZIP8*	*ZIP6 ^ns^*	*ZIP5 ^ns^*
			*ZIP8 ^ns^*	ZIP14*	*ZIP10 ^ns^*	*ZIP8 ^ns^*
			ZIP14*		*ZIP13 ^ns^*	
					*ZIP14 ^ns^*	

(ESCA, Esophageal carcinoma; STAD, Stomach adenocarcinoma; LIHC, liver hepatocellular carcinoma; CHOL, Cholangio carcinoma; PAAD, Pancreatic adenocarcinoma; READ, Rectum adenocarcinoma) All data were obtained from GEPIA2 database.*P < 0.05, ns, not significant (http://gepia2.cancer-pku.cn/#survival).

### The mRNA Levels of *ZIP2/ZIP5/ZIP8*/*ZIP9/ZIP14* Are Decreased in the Liver Tissues of HCC Patients

**Figure 2:**
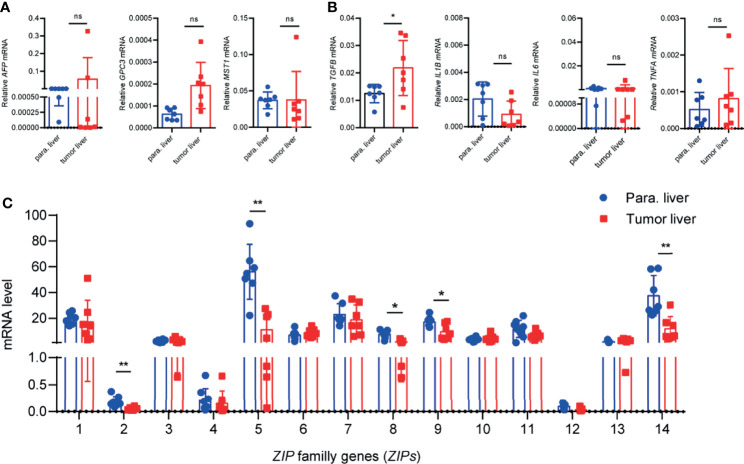
The transcription patterns of *ZIPs* in human liver cancer tissues and paracarcinoma liver tissues. **(A)** The mRNA levels of *AFP*, *GPC3*, *MST1* were detected in human liver cancer tissues and paracarcinoma liver tissues by RT-PCR (n = 9). **(B)** Inflammation-related gene *TGFB*, *IL1B*, *IL6*, *TNFA* were measured in human liver cancer tissues and paracarcinoma liver tissues by RT-PCR (n = 9). **(C)** mRNA abundance profiles of *ZIP*s in human liver cancers and paracarcinoma liver tissues by RT-PCR (n = 9). The small circles represent the individual paracarcinoma liver tissues, and the squares represent the liver cancer tissues. The expressed values were calculated by the 2^−ΔCT^ value. *P < 0.05, **P < 0.01, ns, not significant. The representative data from at least three independent experiments are shown. Data represent mean ± SD. By one-way ANOVA with Newman-Keuls or Holm-Sidak’s multiple comparisons test or Student’s t test.

Since the transcriptions of *ZIPs* in multiple DSCs showed distinct patterns between cancers and healthy controls, then we wondered the individual transcription profile of each *ZIP* in HCC. Next, we collected 9 pairs of cancer and paracarcinoma tissues from patients with HCC. We analyzed the mRNA levels of some HCC related genes, including *AFP*, *GPC3, MST1* and some inflammation-related genes, such as *TGFB*, *IL1B*, *IL6* and *TNFA* by RT-PCR firstly. Interestingly, *TGF-B* transcription was significantly increased in liver cancers compared with paracarcinoma tissues (**Figures 2A, B**). Additionally, liver cancer tissues of HCC patients showed increased numbers and size of tumors compared with paracarcinoma tissues by HE staining of tissue sections (**Supplemental Figure 2**). Then the transcription patterns of *ZIPs* in liver cancers and paracarcinoma tissues were analyzed. The results showed that the mRNA levels of *ZIP2*, *ZIP5*, *ZIP8*, *ZIP9*, and *ZIP14* were significantly lower in liver tumor tissues than those in paracarcinoma tissues (**Figure 2C**). Particularly, the mRNA level of *ZIP5* (approximately 4-fold, P=0.0005) and that of *ZIP14* (approximately 3-fold, P=0.003) in the paracarcinoma tissues of HCC were significantly higher than those in tumor tissues. However, transcription of other *ZIP* genes, such as *ZIP1*, *ZIP3*, *ZIP4*, *ZIP6*, *ZIP7*, *ZIP10*, *ZIP11*, *ZIP12*, had no significant difference between human liver cancers and paracarcinoma tissues (**Figure 2C**). Collectively, all observations demonstrate that *ZIP5*, *ZIP8* and *ZIP9* show the decreased mRNA transcription profiles in cancer tissues of HCC patients.

### The Transcription Profiles of a Group of *Zips* in Liver Cancer Tissues of DEN- and CCl_4_- Induced Mouse Model of HCC Are Decreased

**Figure 3:**
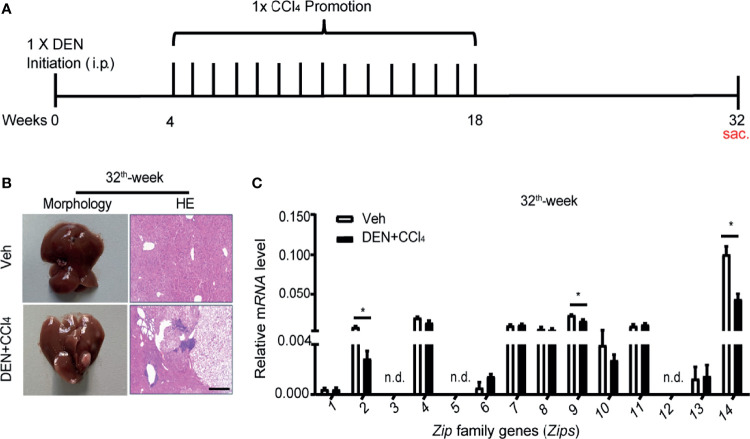
The transcription levels of *Zips* with DEN and CCl_4_- induced HCC model in mice. **(A)** Scheme of the DEN and CCl_4_- induced HCC model. WT male mice were treated with controls (Veh) or DEN (100 mg/kg) to induce HCC. Four-week later, control mice injected with olive oil (0.5 ml/kg; i.p.), whereas DEN treated mice were received CCl_4_ (0.5 ml/kg; i.p.) weekly. **(B, C)** After DEN treated 32 for weeks, the pathological characteristics were presented by morphology and HE staining of section of livers **(B)**. The mRNA levels of *Zips* were detected by RT-PCR **(C)**. Values are mean ± SD from 3 independent experiments. Using 2-tailed, unpaired Student’s t test (n = 7). P values are as followed: *P < 0.05, n.d., not detected.

To further determine whether *Zip* genes were involved in hepatocarcinogenesis, a DEN and CCl_4_- induced mouse model of HCC was established as described by previous studies (35, 36) (**Figure 3A**). Then observation of the mRNA changes of *Zips* in the stage of HCC was performed. At the 32th weeks of DEN injection, which mimicked the occurrence stage of HCC (37, 38), tumors were clearly observed on the surface of the livers. HE staining of liver sections showed an increased nuclear-to-cytoplasmic ratio of cancer cells. The normal hepatic lobular structure was completely destroyed in liver sections of these mice (**Figure 3B**). By FACS analysis using anti-F4/80+ antibody, we confirmed the accumulation of intrahepatic macrophages in DEN-induced mice compared with vehicle-treated mice (**Supplemental Figure 1**). Furthermore, the mRNA levels of *Zip*s, in the liver tissues of these mice were measured by RT-PCR, the results showed that *Zip14* gene transcription was down-regulated, which was consistent with bioinformatics data (**Figure 1**). Besides, the mRNA levels of *Zip2* and *Zip9* were also down-regulated, which was consistent with the transcriptional pattern data of clinical HCC samples (**Figures 2C**, **3C**). In summary, *Zip2*, *Zip9*, and *Zip14* transcriptions were dramatically decreased in cancer tissues from DEN and CCl_4_- induced mice model of HCC.

### The mRNA Levels of *Zips* Are Changed in M1 or M2 Polarized Macrophages

**Figure 4:**
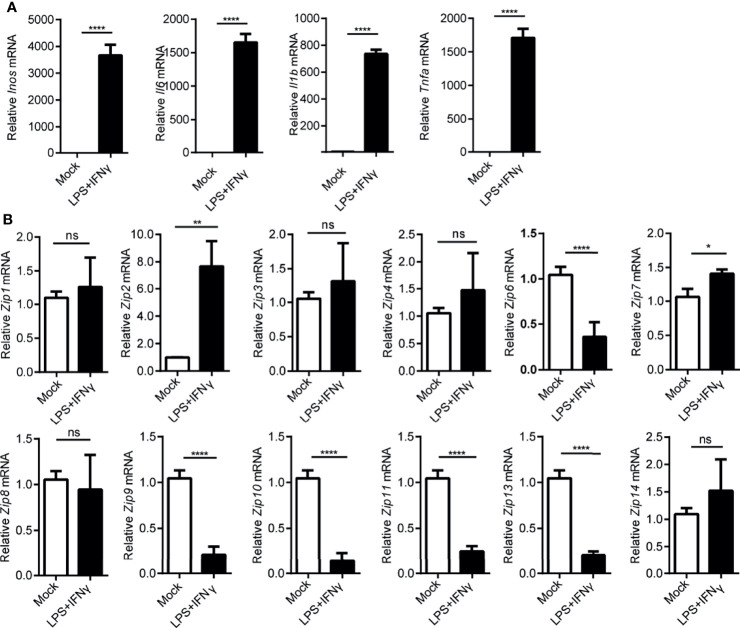
Transcription of *Zip9* is downregulated during M1 macrophage polarity. **(A)** M1 macrophage characteristic genes were assessed by RT-PCR. M1 macrophages were generated by PEMs stimulating with LPS plus IFNγ. Data were normalized for β-actin mRNA levels. Results were presented as the average ± SD of three independent experiments. **(B)** PEMs were stimulated with LPS plus IFNγ for 6 h to measure *Zips* mRNA levels by RT-PCR. Results were presented as the average ± SD of three independent experiments. Data were normalized for β-actin mRNA levels. A, B was analyzed by Student’s t-test. The representative data from three independent experiments are shown. P values are as followed: *P < 0.05, **P < 0.01, and ****P < 0.0001, ns, not significant.

**Figure 5:**
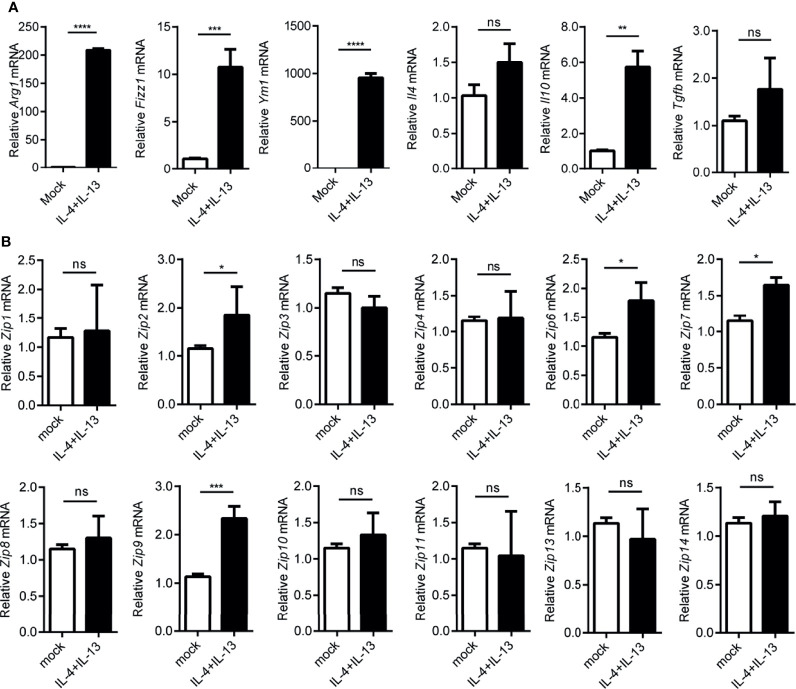
Transcription of *Zip9* is upregulated during M2 macrophage polarity. **(A)** The mRNA levels of *Arg1*, *Ym-1*, *Fizz1*, and *Il10*, *Il4*, *Tgfb* were detected in IL-4 and IL-13 (20 ng/ml each) stimulated M2-polarized macrophages. **(B)** *Zip*s transcription was detected by RT-PCR. PEMs were stimulated with IL-4 and IL-13 for 24 h. Data are shown as means ± S.D (n = 4). Data were normalized for β-actin mRNA levels. A, B was analyzed by two-tailed Student’s t-test. The representative data from three independent experiments are shown. P values are as followed: *P < 0.05, **P < 0.01, ***P < 0.001 and ****P < 0.0001, ns, not significant.

Since macrophages were suggested to play an important role in hepatocarcinogenesis previously (35), as reported before, we observed the infiltration of monocyte-derived macrophages in liver cancers by FACS analysis (**Supplemental Figure 1**). A large amount of CD68 positive macrophages were found in paracarcinoma tissue and liver cancers (**Supplemental Figure 2**). Given that macrophages could be typically categorized into M1 or M2 phenotypes by adapting to local microenvironment during the progress of HCC, we wondered whether *Zips* were involved in the polarization process of these macrophages. To address this, firstly, LPS plus IFNγ were added to the culture medium of the peritoneal elucidated macrophages (PEMs) to induce M1-polarized macrophages *in vitro.* Interestingly, the mRNA levels of M1 marker genes including *Il6*, *Inos*, *Il1b* and *Tnfa* were significantly augmented in PEMs (**Figure 4A**). These data strongly indicated that macrophages were polarized towards M1 phenotype. We then analyzed the transcription levels of *Zips* by RT-PCR in M1 phenotype macrophages. We observed that *Zip2* and *Zip7* mRNA were significantly increased in M1 macrophages (the mRNA of *Zip2* was 7-fold higher than that in the untreated PEMs, P =0.032). Moreover, the mRNA levels of *Zip6*, *Zip9*, *Zip10*, *Zip11*, and *Zip13* were comparable in M1 macrophages. But *Zip5* and *Zip12* were not detected in M1 macrophages (**Figure 4B**). We next detected the mRNA levels of *Zips* in M2-polarized macrophages. Under IL-4 and IL-13 stimulation, the mRNA levels of M2 marker genes including *Ym1*, *Arg1*, *Fizz1*, *Il10*, *Il4* and *Tgfb* were detected. *Ym1*, *Arg1*, *Fizz1* and *Il10* mRNA expression were significantly increased in the M2- polarized macrophage cells compared with untreated cells (**Figure 5A**). Similarly, we also analyzed the transcription levels of *Zips* by RT-PCR in M2-polarized macrophages. The transcription levels of *Zip2*, *Zip6*, *Zip7*, *Zip9*, were elevated in M2 phenotype macrophages significantly. However, there were no significant differences in the mRNA levels of *Zip1*, *Zip3*, *Zip4*, *Zip8*, *Zip10*, *Zip11*, *Zip13*, and *Zip14* (**Figure 5B**). Taken together, our data show that the mRNA levels of *Zips* are differently changed in M1 or M2 polarized macrophages.

### The Transcription of *ZIP9* Is Decreased in Human TAMs From HCC Patients

**Figure 6:**
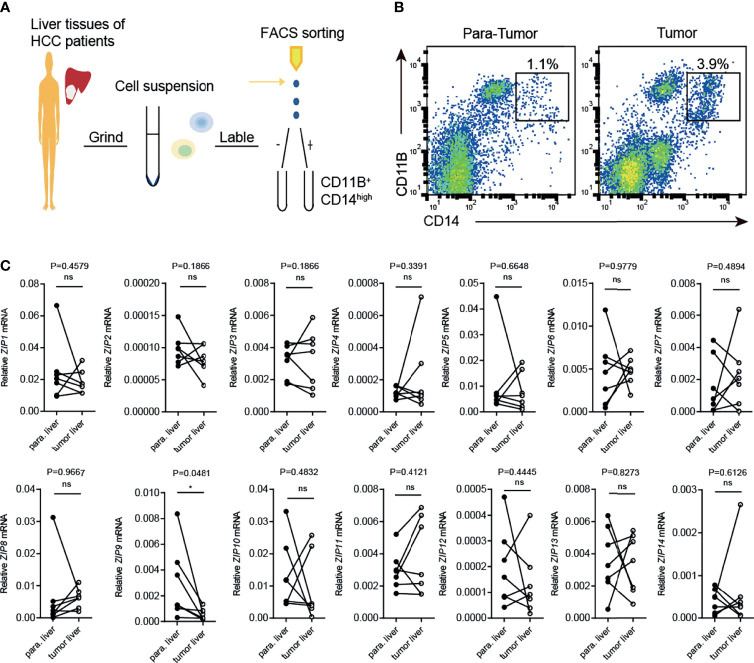
Transcription of *Zip9* is downregulated in HCC patients-derived TAMs. **(A)** FACS protocol was used for isolating HCC patients-derived TAMs. **(B)** Human liver cancer tissues and paracarcinoma liver tissues were digested, stained for CD14, CD11B and then analyzed by flow cytometry. **(C)** The mRNA levels of *ZIPs* in TAMs isolated from human liver cancer tissues and paracarcinoma liver tissues were detected by RT-PCR (n = 7). The black points represent the individual paracarcinoma liver tissue, and the black circles represent the liver cancer tissue. The expressed values were calculated by the 2^−ΔCT^ method. *P < 0.05, ns, not significant. The representative data from at least three independent experiments are shown. Data were determined with 2-tailed unpaired Student’s t test between 2 groups.

To further confirm the transcriptional alternation of *ZIP9*, flow cytometry was used to sort tumor-associated macrophages (CD11B^+^ CD14^high^ TAMs) generated from tumor liver tissues (tumor-TAMs) and paracarcinoma tissues (para-TAMs) of HCC patients. The process was shown in **Figure 6A**. The results showed that the percentage of tumor-TAMs was increased, as compared with para-TAMs (**Figure 6B**). The transcription levels of *ZIPs* were further verified by RT-PCR in these two patients-derived TAMs: tumor-TAMs and para-TAMs. Notably, a decrease in *ZIP9* transcription was observed in tumor-TAMs. However, transcription of other *ZIP* genes, such as *ZIP2*, *ZIP5*, *ZIP8*, *ZIP10*, *ZIP11*, *ZIP12*, had no significant difference between tumor-TAMs and para-TAMs (**Figure 6C**). These findings indicate that *ZIP9* is transcriptionally downregulated not only in HCC patient-derived TAMs but also in human liver cancer tissues, which suggests that *ZIP9* may be involved in hepatocarcinogenesis by participating in TAMs differentiation.

### *Zip9* Participates in Macrophage Polarization *In Vitro*

**Figure 7:**
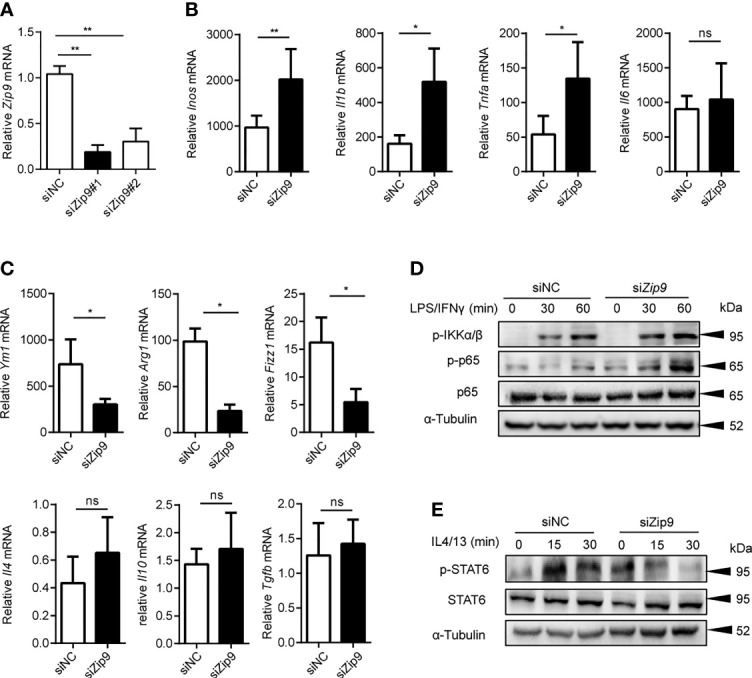
*Zip9* modulates the macrophage polarity. **(A)** The siRNA knockdown efficiencies of *Zip9*. *Zip9* was analyzed by RT-PCR following transfection with *Zip9* siRNA 48 hours. **(B)** The analysis of M1-polarized macrophages characteristic genes (*Inos*, *Il6*, *Il1b*, *Tnfa*). Genes were detected by RT-PCR, following transfection with control small interfering RNA (siRNA) or *Zip9* siRNA and stimulated with LPS plus IFNγ. **(C)** The analysis of M2-polarized macrophages characteristic genes (*Ym1*, *Arg1*, *Fizz1*, *Il10*, *Il4*, *Tgfb*). Genes were detected by RT-PCR, following transfection with control siRNA or *Zip9* siRNA and stimulated with IL-4 and IL-13 (20 ng/ml each) *P < 0.05, **P < 0.01 and ns, not significant. Data were analyzed by Student’s t-test. Results were presented as the average ± SD of at least three independent experiments. **(D)** PEMs were pretreated with control small interfering RNA (siRNA) or *Zip9* siRNA for 48h followed by 30/60 mins stimulation with IFNγ and LPS. Cell lysates were processed for immunoblotting with antibody against phospho-IκBα/β and phospho-p65 followed by stripping and reprobing with antibody against α-tubulin. **(E)** PEMs were pretreated with control small interfering RNA (siRNA) or *Zip9* siRNA for 48h followed by 15/30 mins stimulation with IL-4 and IL-13. Cell lysates were processed for immunoblotting with antibody against phospho-STAT6 and total-STAT6 followed by stripping and reprobing with antibody against α-tubulin. Each panel is a representative experiment of at least three independent biological replicates.

Above all, the results showed that *ZIP2* (*Zip2*) and *ZIP9* (*Zip9*) were significantly decreased in both cancer tissues of human HCC and mice HCC. In addition, the mRNA levels of *Zip2* and *Zip9* had significant changes in the polarized macrophages compared with native macrophages (**Supplemental Figure 3A**). These observations indicated that *Zip2* and *Zip9* might be involved in macrophages polarization. To investigate their roles in these processes, specific siRNA was used to knock down *Zip2* or *Zip9* in mouse PEMs, and the knockdown efficiency was confirmed by RT-PCR analysis (**Figure 7A** and **Supplemental Figure 3B**). The knockdown of *Zip9* (Si*Zip9*) significantly enhanced the transcription of M1 macrophage markers, such as *Inos*, *Il1b* and *Tnfa* (**Figure 7B**). However, reduced mRNA levels of M2 macrophage markers, such as *Ym1*, *Arg1*, *Fizz1* were detected by RT-PCR in *Zip9*-specific knockdown macrophages (**Figure 7C**). Knockdown of *Zip2* significantly promoted macrophages toward M1 polarized differentiation while knockdown of *Zip2* had no changes in M2 polarization (**Supplemental Figures 3C, D**). Our data showed that *Zip9*, but not *Zip2*, could inhibit M1 macrophage polarization in response to LPS and IFNγ treatment *in vitro* and promote M2 macrophage polarization in response to IL-4 plus IL-13 stimulation *in vitro*. It had been reported that Zip8 could regulate the phosphorylation of IKK-β to attenuate the NF-κB signaling pathway (39), and increased cytosolic Zn^2+^ promotes the phosphorylation of STAT6 to enhance the anti-inflammation effects of IL-4 (40). To clarify the mechanism of how *Zip9* is involved in regulating macrophage polarization, we determined the expression of signaling effectors of the NF-κB in M1 macrophages and IL4/STAT6 in M2 macrophages after *Zip9* siRNA knockdown. As demonstrated, the levels of phosphorylation of IκBα/β (p-IκBα/β) and p65 (p-p65) were increased in the Si*Zip9* group following LPS plus IFNγ stimulation (**Figure 7D**) while phosphorylated STAT6 (p-STAT6) was significantly inhibited by *Zip9* siRNA in IL-4 and IL-13 treated PEMs (**Figure 7E**). Taken together, our results indicate that *Zip9* likely plays a role in classical M1/M2 polarization of macrophages. Zip9 inhibits M1 polarization by reducing phosphorylation of IκBα/β pathways while promoting M2 polarization by increasing phosphorylation of STAT6.

## Discussion

In this study, we first analyzed the mRNA transcription profiles of *ZIPs* in digestive system cancers by bioinformatics, and revealed the significant changes of these gene transcripts between cancers and healthy controls (**Figure 1**). Previous reports showed that ZIPs were involved in the pathology of various digestive system cancers in clinical analysis and experimental investigations. Costello et al. performed immunological staining on normal pancreas and pancreatic sections and found that ZIP3 was significantly reduced in pancreatic cancer tissues (17). Li et al. observed the expression of ZIP4 in 17 clinical pancreatic cancer samples, and found that ZIP4 was significantly overexpressed in 16 samples compared with surrounding normal tissues, and the expression of *ZIP4* mRNA in human pancreatic cancer cells was significantly higher than that in human pancreatic duct epithelium cells, indicating that up-regulation of ZIP4 may be involved in the pathogenesis and progression of pancreatic cancer (19). Jin et al. also showed that exogenous ZIP4 can promote the growth of pancreatic cancer through subcutaneous BALB/c nude mouse models *in vivo* and statistics of clinical data, which indicated ZIP4 can be a new diagnostic biomarker for pancreatic cancer (11). Sheng et al. tested the expression of ZIP7 in human colorectal cancer and five colorectal cancer cell lines, they showed that the expression level of ZIP7 in colorectal cancers was higher than that in normal colon tissues, and when the *ZIP7* gene was knocked-down on colorectal cancer cells, the viability and proliferation ability of colorectal cancer cells were significantly decreased. Therefore, they speculated that ZIP7 may be a potential oncogene, which played an important role in the occurrence and development of colorectal cancer (12). These investigations were consistent with our observations (**Figure 1**). We hypothesize that *ZIPs* might play roles in the pathogenesis and progression of liver cancers. However, the relevant research about total transcription pattern of *ZIPs* in HCC is not clear.

In fact, it has been shown that the zinc element in the peripheral blood and liver cancer tissues of LIHC patients is significantly reduced (5). Small-scale clinical trials reported that long-term zinc supplementation can help reduce the occurrence of liver cancer (41). ZIPs are responsible for the absorption of zinc in the body (10). Therefore, we speculate that the abnormal absorption of zinc in LIHC due to the abnormal transcription of the *ZIPs* affects the body’s immune defensive response. In order to further test our hypothesis, nine matched samples of LIHC patients’ cancer and para-cancerous tissues were collected for detecting *ZIPs* mRNA levels. The results showed that the mRNA levels of *ZIPs* in cancerous and para-cancerous tissues were different. Besides previously reported *ZIP14* (42), the transcription levels of *ZIP2*, *ZIP5*, *ZIP8* and *ZIP9* were also found a substantial decrease in liver cancer tissues (**Figure 2C**). By establishing DEN and CCl_4_ **-** induced mouse model of HCC, we showed that *Zip2*, *Zip9* and *Zip14* among all the families were down-regulated in liver tissues of the mice with HCC induction (**Figure 3C**). The clues are worthy of further in-depth research on their roles in hepatocarcinogenesis through genetically engineered mouse models.

The involvement of macrophages in the development of HCC has been reported (43). The polarization of macrophages into type-1 or type-2 had different effects on the occurrence and development of HCC (22). Li et al. reported that the decrease of MST1 is beneficial to the occurrence and development of HCC (35). Furthermore, we are curious about whether *Zips* are involved in the regulation of macrophage polarity. Interestingly, our observations with both patient samples and mouse models demonstrated a large number of macrophages infiltrated in the liver cancer tissues (**Supplemental Figures 1, 2**). Notably, macrophages have been suggested to play an essential role in inflammatory microenvironment. The involvement of *ZIP* members in macrophage polarity was also shown before. THP-1 monocytes or macrophages showed zinc deficiency by adding zinc chelating agent TPEN *in vitro*, the expression of *ZIP1* did not change, whereas the mRNA of *ZIP2* gene was significantly increased (34). In addition, the phagocytic function of the THP1 cells with *ZIP7* knockdown was severely inhibited, and further restored by supplementing exogenous Zn^2+^ (44). The expression of M2-macrophages polarization marker CD206 in zip7-deficient macrophages was increased, while the expression of M1 marker NOS2 was decreased (44). ZIP8 was also reported to be stimulated by LPS and TNFα, resulting in a rapid increase in intracellular zinc levels (39). Accordingly, increased zinc concentration negatively regulated NF-κB by inhibiting the IκB kinase β subunit (IKKβ) in the kinase domain, thereby inhibiting inflammation and increasing the survival rate of immune cells (39). Mechanistically, NF-κB was also reported to regulate the expression of ZIP8 in monocytes, macrophages, dendritic cells and lung epithelial cells. In innate immunity, the deficiency of ZIP10 could reduce the number of macrophages and monocytes in the inflammatory response induced by LPS, which is caused by an increase in P53-dependent mortality (45). Using LPS to stimulate human primary macrophages, it was found that the mRNA transcription level of *ZIP14* was up-regulated (46). All these studies have shown that the involvement of ZIPs in the function of macrophages. More interestingly, in our study, we found the transcription levels of *Zips* were differentially regulated during the polarization of macrophages. Then the transcription patterns of *ZIPs* on human liver cancer-derived TAMs were detected. The results showed that *ZIP9* was down transcription in tumor-TAMs compared with para-TAMs. Here, we demonstrated that *Zip9* upregulation macrophage M2 polarization *via* IL4/STAT6 pathway and downregulation macrophage M1 polarization *via* IκBα/β-p65 pathway mechanistically. Further investigation on the crosstalk between these signaling events in the cytosol of macrophages is needed to uncover the detailed molecular mechanism for the purpose of discovering new biomarkers for the diagnosis of HCC in the future.

## Data Availability Statement

The raw data supporting the conclusions of this article will be made available by the authors, without undue reservation.

## Ethics Statement

The studies involving human participants were reviewed and approved by Shanghai Zhongshan Hospital ethics committee. The patients/participants provided their written informed consent to participate in this study. The animal study was reviewed and approved by Ethics Committee of Shanghai University.

## Author Contributions

BW conceptualized and designed the study. YG and DY contributed to the conceptual design, writing, editing, and generation of figures for this manuscript. TT, XZ, RZ, JR, DT, YM, HZ, YL and YW participated in the experimental work. YG, DY and BW wrote the manuscript. All authors read and approved the final manuscript.

## Funding

This work was supported by grants from the Ministry of Science and Technology of China (2016YFD0500207), the National Natural Science Foundation of China (81961160738, 81825011, 81571617 and 81930038) and the Strategic Priority Research Program of the Chinese Academy of Sciences (XDB19030200).

## Conflict of Interest

The authors declare that the research was conducted in the absence of any commercial or financial relationships that could be construed as a potential conflict of interest.

## Publisher’s Note

All claims expressed in this article are solely those of the authors and do not necessarily represent those of their affiliated organizations, or those of the publisher, the editors and the reviewers. Any product that may be evaluated in this article, or claim that may be made by its manufacturer, is not guaranteed or endorsed by the publisher.
