# Supplementary material for: The Transcription of ZIP9 Is Associated With the Macrophage Polarization and the Pathogenesis of Hepatocellular Carcinoma
Source: Front Immunol. 2022 Mar 15;13:725595. doi: 10.3389/fimmu.2022.725595 (PMC8967370; doi:10.3389/fimmu.2022.725595)
Supplement: Supplementary file 1 [file DataSheet_1.pdf]

Supplementary Material

1 Supplementary Figures and figure legend

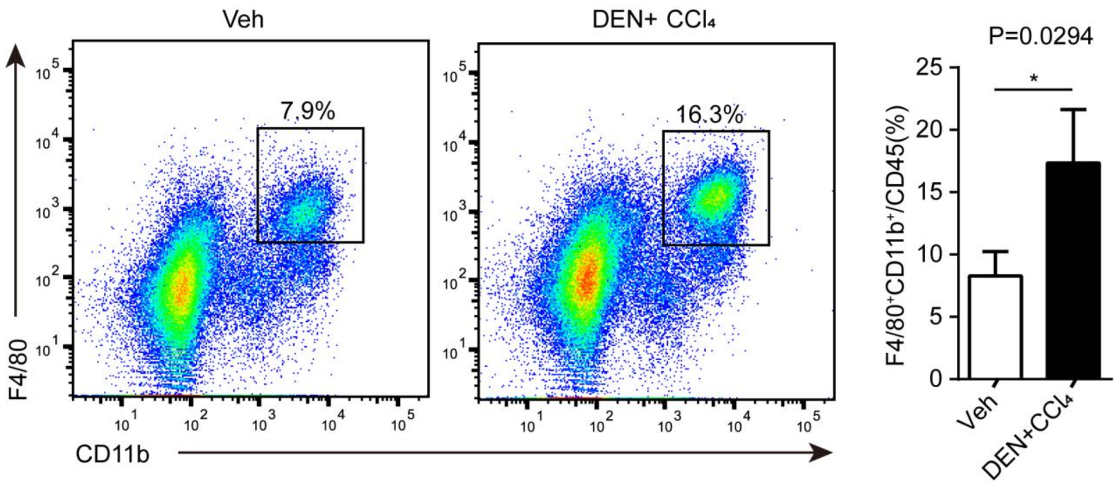

Supplementary Figure 1. More macrophages infiltrate into liver tissues in DEN-induced mouse model of HCC

WT male mice were treated with controls (Veh) or DEN (100 mg/kg) to induce HCC and were sacrificed 32 weeks after DEN injection (n = 3). Percentages of intrahepatic MoMs (F4/80^{+}CD11b^{hi}) were analyzed by FACS (left panel). Data are from three independent experiments mean ± SD (right panel). *P < 0.05, using a two-tailed, unpaired Student's t test.

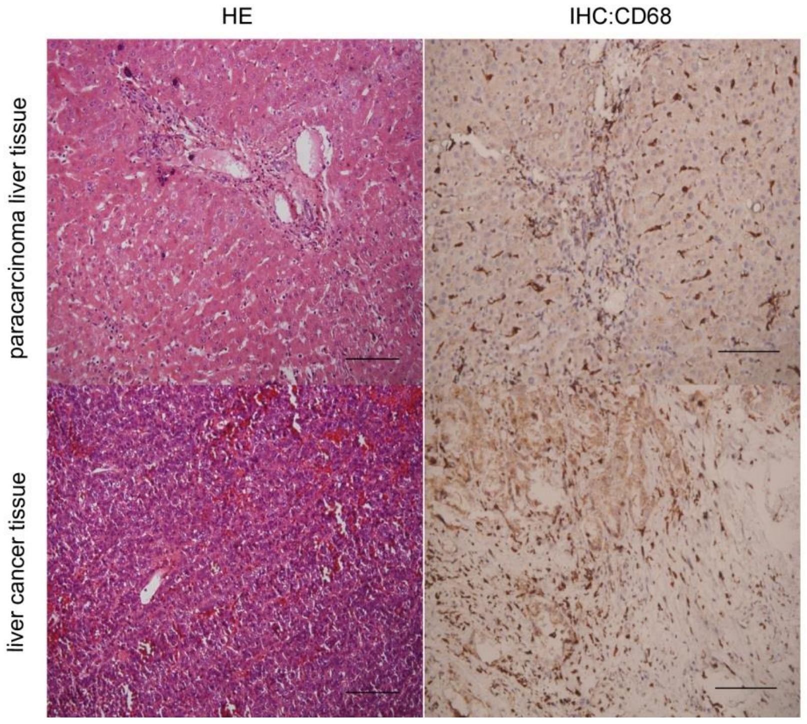

Supplementary Figure 2. More macrophages infiltrate with liver tissues in human HCC

HE staining (scale bar: 250 μm) and IHC staining (scale bar: 250 μm) with anti-CD68 of human liver sections from paracarcinoma tissue and tumors of HCC patients.

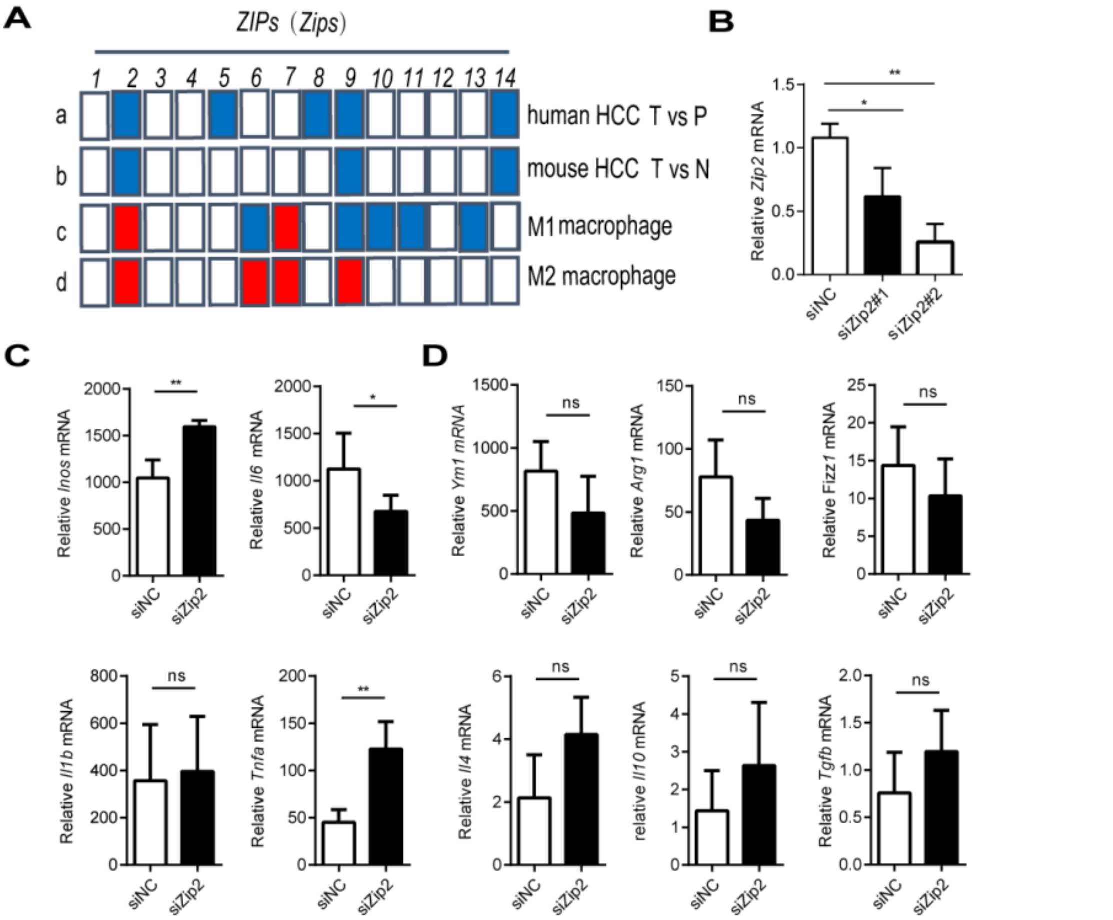

Supplementary Figure 3. ***Zip2*** **knockdown has no effect on M2 macrophages polarity**

(A) Heat-map of transcription changes of *ZIPs/Zips*. a, *ZIPs* changed significantly in human liver cancer tissues and paracarcinoma tissues. b, significantly changed *Zips* in the HCC stage of the mouse model. c, significantly changed *Zips* in M1-polarized macrophages. d, significantly changed *Zips* in M2-polarized macrophages. The red color means up-regulated and blue color represents down-regulated transcription.

(B) The siRNA knockdown efficiencies of *Zip2*. *Zip2* were analyzed by RT-PCR following transfection with *Zip2* siRNA.

(C) M1-polarized macrophages characteristic genes (*Inos*, *Il6*, *Il1b*, *Tnfa*) were detected by RT-PCR following transfection with control small interfering RNA (siRNA) and *Zip2* siRNA then stimulated with LPS plus IFNγ.

(D) M2-polarized macrophages characteristic genes (*Ym1, Arg1, Fizz1, Il10, Il4, Tgf-b*) were detected by RT-PCR following transfection with control small interfering RNA (siRNA) and *Zip2* siRNA then stimulated with IL-4 and IL-13 (20 ng/ml

each).Student's t test. Results were presented as the average ± SD of three independent experiments. *P < 0.05, **P < 0.01 and ns, not significant.
